# Repeated oral dosing of TAS-102 confers high trifluridine incorporation into DNA and sustained antitumor activity in mouse models

**DOI:** 10.3892/or.2014.3487

**Published:** 2014-09-17

**Authors:** NOZOMU TANAKA, KAZUKI SAKAMOTO, HIROYUKI OKABE, AKIO FUJIOKA, KEISUKE YAMAMURA, FUMIO NAKAGAWA, HIDEKI NAGASE, TATSUSHI YOKOGAWA, KEI OGUCHI, KEIJI ISHIDA, AKIKO OSADA, HIROMI KAZUNO, YUKARI YAMADA, KENICHI MATSUO

**Affiliations:** 1 Taiho Pharmaceutical Co., Ltd., Tsukuba Research Center, Tsukuba, Ibaraki 300-2611, Japan; 2 Taiho Pharmaceutical Co., Ltd., Tokushima Research Center, Ebisuno, Hiraishi, Kawauchi-cho, Tokushima 771-0194, Japan; 3 Kanazawa University, Cancer Research Institute, Division of Molecular Virology and Oncology, Kakuma-machi, Kanazawa, Ishikawa 920-1192, Japan

**Keywords:** TAS-102, trifluridine, tipiracil hydrochloride, Lonsurf

## Abstract

TAS-102 is a novel oral nucleoside antitumor agent containing trifluridine (FTD) and tipiracil hydrochloride (TPI). The compound improves overall survival of colorectal cancer (CRC) patients who are insensitive to standard chemotherapies. FTD possesses direct antitumor activity since it inhibits thymidylate synthase (TS) and is itself incorporated into DNA. However, the precise mechanisms underlying the incorporation into DNA and the inhibition of TS remain unclear. We found that FTD-dependent inhibition of TS was similar to that elicited by fluorodeoxyuridine (FdUrd), another clinically used nucleoside analog. However, washout experiments revealed that FTD-dependent inhibition of TS declined rapidly, whereas FdUrd activity persisted. The incorporation of FTD into DNA was significantly higher than that of other antitumor nucleosides. Additionally, orally administered FTD had increased antitumor activity and was incorporated into DNA more effectively than continuously infused FTD. When TAS-102 was administered, FTD gradually accumulated in tumor cell DNA, in a TPI-independent manner, and significantly delayed tumor growth and prolonged survival, compared to treatment with 5-FU derivatives. TAS-102 reduced the Ki-67-positive cell fraction, and swollen nuclei were observed in treated tumor tissue. The amount of FTD incorporation in DNA and the antitumor activity of TAS-102 in xenograft models were positively and significantly correlated. These results suggest that TAS-102 exerts its antitumor activity predominantly due to its DNA incorporation, rather than as a result of TS inhibition. The persistence of FTD in the DNA of tumor cells treated with TAS-102 may underlie its ability to prolong survival in cancer patients.

## Introduction

Colorectal cancer (CRC) is the fourth most common cause of cancer-related mortality (1). The main treatments for patients with advanced metastatic colorectal cancer (mCRC) include systemic combination chemotherapies (2–7). Although the standard therapies are initially effective, many patients relapse due to the onset of drug resistance and are subsequently placed on salvage chemotherapy.

TAS-102 is a novel oral nucleoside antitumor agent that consists of trifluridine (FTD) and tipiracil hydrochloride (TPI) at a molar ratio of 1:0.5. FTD is the antitumor component of TAS-102, whereas TPI prevents degradation of FTD through a first-pass effect as a thymidine phosphorylase inhibitor. FTD is a well-known antiproliferative agent with two mechanisms of action; it inhibits thymidylate synthase (TS) and is also incorporated into DNA (8,9). Recently, TAS-102 was found to significantly improve overall survival of mCRC patients in whom systemic chemotherapy is either ineffective or not tolerated (10).

Since the serum half-life of FTD is relatively short after intravenous (i.v.) injection, split dose regimens, as well as single rapid bolus injections, have been used in the clinic (11). However, i.v. injection of FTD has been discontinued due to disease recurrence and prolonged toxicity (12), and the drug is now currently used as an ophthalmic solution for treatment of epithelial keratitis caused by the herpes simplex virus (13).

FTD instability in serum is a result of degradation by thymidine phosphorylase (TP), and therefore co-administration of TPI with FTD is employed to increase the effective *in vivo* FTD concentration (14). We previously developed a TAS-102 anticancer treatment regimen in which FTD was orally administered and combined with TPI; this approach significantly prolonged survival.

These clinical results suggest that the mechanism of action of orally administered FTD and TPI co-treatment may differ from that of i.v. administration of FTD alone. We hypothesized that the balance between TS inhibition and DNA incorporation may be altered according to the FTD administration mode. Therefore, we examined both delivery routes in comparative studies to determine the mechanism by which TAS-102 exerts its antitumor activity.

## Materials and methods

### Chemicals and reagents

FTD was obtained from Yuki Gosei Kogyo Co., Ltd. (Tokyo, Japan). 5-fluoro-2′-deoxyuridine (FdUrd) was obtained from Wako Pure Chemical Industries (Osaka, Japan). TPI was synthesized at Junsei Chemical (Tokyo, Japan). Tegafur (FT), gimeracil (CDHP) and oteracil potassium (Oxo) were synthesized in-house at Good Manufacturing Practice grade. Cytarabine (Ara-C) was obtained from Sigma-Aldrich K.K. (Tokyo, Japan). Gemcitabine (dFdC) was synthesized in-house. [6-^3^H] FTD (10.0 Ci/mmol), [6-^3^H] FdUrd (13.5 Ci/mmol), [5^−3^H] Ara-C (26.7 Ci/mmol) and [5-^3^H] dFdC (26.0 Ci/mmol) were obtained from Moravek (Brea, CA, USA).

### Animals

Five-week-old male nude mice (BALB/cA Jcl-*nu/nu*) were purchased from CLEA Japan Inc. (Tokyo, Japan) and maintained in accordance with the animal experimental regulations of Taiho Pharmaceutical Co., Ltd. Mice were allowed free access to a commercial diet and water (filtered and chlorinated) and exposed to a 12-h light/12-h dark cycle in a barrier facility.

### Cell culture and human cancer xenografts in nude mice

Eight human cancer xenografts were used (CRC cell lines DLD-1, HT-29, HCT116, KM20C and SW480; small cell lung cancer cells Lu-134; breast cancer cells MC-2 and MX-1). Lu-134, MC-2 and MX-1 were maintained by serial transplantation in the dorsum of nude mice. Other xenografts were established by implantation of cultured cells before each study. Lu-134 and MC-2 were obtained from the Central Institute for Experimental Animals (Tokyo, Japan). NUGC-3, HT-29 and MX-1 cells were provided by the Japanese Foundation for Cancer Research (Tokyo, Japan). DLD-1 and HCT116 cells were obtained from Dainippon Pharmaceutical (Tokyo, Japan). KM20C cells were kindly provided by the National Cancer Center (Tokyo, Japan). SW480 cells were purchased from American Type Culture Collection (Manassas, VA, USA). HeLa cells were obtained from the Health Science Research Resources Bank (Tokyo, Japan). These cultured cells were maintained at 37°C with 5% CO_2_, in the culture medium recommended by each provider.

### Evaluation of growth inhibitory effect in vitro

HeLa cells were seeded in 96-well plates at 500 cells/180 μl/well in triplicate, pre-cultured for 24 h, and then 20 μl of each drug solution was added for 24 or 72 h. For the 24-h treatment, cells were washed with phosphate-buffered saline (PBS) after treatment, drug-free medium was added to each well, and the culture was further incubated for 48 h. Cell growth inhibition was evaluated using a Cell Counting Kit-8 (Dojindo Laboratories) according to the manufacturer’s instructions. The 50% inhibitory concentration (IC_50_) values were calculated from the absorbance data using SAS (SAS Institute, Cary, NC, USA).

### Measurement of dTTP in vitro

Cells were seeded in 175-cm^2^ culture flasks at 1×10^6^ cells and pre-cultured for 24 h, prior to treatment with test compounds. After each treatment, cells were collected and resuspended in 100 μl PBS, and 50 μl of 1.26 N perchloric acid (PCA) was added to the cell suspension. The mixture was left on ice for 10 min and centrifuged for 5 min at 15,000 rpm. Three hundred microliters of 0.5 N tri-n-octylamine dissolved in CH_2_Cl_2_ was added to the supernatant and centrifuged for 5 min at 15,000 rpm. The supernatant was applied to an HPLC system (Prominence, Shimadzu Corporation, Kyoto, Japan) equipped with an ODS reverse phase column (250×4.6 mm, 5 μm; Phenomenex), under an analytical method utilizing eluent A [10 mM KH_2_PO_4_, 10 mM tetrabutylammonium hydroxide (TBA-OH), 0.25% methanol, pH 8.0], and eluent B (50 mM KH_2_PO_4_, 5.6 mM TBA-OH, 50% methanol, pH 6.5) at a solvent flow rate of 0.80 ml/min in a 120-min gradient elution as follows: B 60% from 0 to 10 min, B 60–70% from 10 to 20 min, B 70% from 20 to 45 min, B 70–80% from 45 to 55 min, B 80% from 55 to 80 min, B 80–100% from 80 to 90 min, B 100% from 90 to 100 min, B 100-60% from 100 to 101 min, and B 60% from 101 to 120 min.

### Evaluation of the nucleoside analogue levels incorporated into DNA in vitro

After each drug treatment, 1×10^6^ cells were harvested and DNA was extracted with a DNeasy Tissue kit (Qiagen GmbH, Hilden, Germany). The DNA solution was dissolved in 10 ml of liquid scintillation fluid (ACS-II; GE Healthcare) to measure the radioactivity, and the quantity of each tritium-labeled form in the DNA was calculated. The DNA concentration was calculated based on the absorbance at 260 nm.

### In vivo evaluation of antitumor activity in mice xenograft models

For experiments, ~8 mm^3^ cubic fragments of tumor were implanted subcutaneously into the axilla of mice. For preparation of the orally administered FTD solution, FTD was dissolved in a 0.5% aqueous solution of hydroxypropyl methyl-cellulose (HPMC; Shin-Etsu Chemical Co., Ltd., Tokyo, Japan). The FTD solution for continuous infusion was prepared by dissolving FTD in physiological saline. The TAS-102 dosing solution was composed of FTD and TPI dissolved in a 0.5% aqueous solution of HPMC at a molar ratio of 1:0.5. The dose of TAS-102 was expressed on the basis of the amount of FTD. For preparation of the S-1 solution, FT, CDHP and Oxo were dissolved in a 0.5% aqueous solution of HPMC at a molar ratio of 1:0.4:1. The dose of S-1 was expressed on the basis of the amount of FT. When the tumor volume reached ~100 mm^3^, the mice were randomly assigned into different treatment groups. Nude mice in the treatment group received the test compounds. The control group received no treatment. For continuous infusion, the compound was administered with an osmotic pump (ALZET osmotic pump model 1002; DURECT Corporation, Cupertino, CA, USA). Tumor volume was measured twice a week throughout the experiments. Tumor volume and relative tumor volume were calculated according to the following formula: Tumor volume = (width)^2^ × (length)/2; and relative tumor volume = (mean tumor volume during treatment)/(mean tumor volume at the start of treatment). The following formula was used to calculate the antitumor effect: Tumor growth inhibition rate (%) = 1 - (mean relative tumor volume of the treatment groups/the mean relative tumor volume of the control group). Body weight changes (BWCs) were used as a proxy measure of side-effects, and calculated according to the following formula: BWC (%) = [(BW on evaluation date) - (BW on grouping date)]/(BW on day grouping date) × 100. The tumor growth inhibition rate (IR) % on day 15 was used to quantify the antitumor effect. In parallel, some tumors were isolated, immersed in liquid nitrogen, and stored at −80°C for additional experiments described below.

### Analysis of FTD incorporation into the DNA of tumor tissues in mouse xenografts

DNA was extracted from tumors using the DNA Isolation kit for Cells and Tissues (Roche Diagnostics GmbH, Penzberg, Germany). The extracted DNA was diluted to 500 μg/ml with ddH_2_O and degraded to nucleosides in a 200-μl reaction mixture consisting of 100 mM Tris-HCl (pH 7.0), 50 mM NaCl, 2.5 mM CaCl_2_, 10 mM MgCl_2_, 1 U DNaseI (Takara Bio Inc., Tokyo, Japan), 40 μg phosphodiesterase I (Sigma-Aldrich, St. Louis, MO, USA), 2 U alkaline phosphatase (Nippon Gene Co., Ltd., Tokyo, Japan) and 10 μg extracted DNA at 37°C for 2 h. After the reaction, 20 μl of 4.2 N PCA was added to each reaction mixture and centrifuged at 15,000 rpm for 3 min. Sixty microliters of 1 M K_2_HPO_4_ was added to each sample for neutralization and centrifugation was performed to remove debris. The supernatant was analyzed by HPLC system with ODS reverse phase column (250×4.6 mm, 5 μm; Phenomenex, Shimadzu GLC Ltd., Tokyo, Japan), under the analytical method with eluent A [10 mM sodium phosphate (pH 6.8), 5% CH_3_CN], eluent B [10 mM sodium phosphate (pH 6.8), 60% CH_3_CN], at a solvent flow rate of 1.0 ml/min in a gradient elution as follows: B 0% from 0 to 5 min, B 0–20% from 5 to 10 min, B 20% from 10 to 15 min, B 20–70% from 15 to 20 min, B 70% from 20 to 25 min, B 70-0% from 25 to 25.1 min, and B 70-0% from 25.1 to 35 min.

### Measurement of deoxyuridine monophosphate (dUMP) in tumor tissues of mouse xenografts

Collected tumors were homogenized with 0.48 N PCA and centrifuged for 5 min at 2,000 rpm. Supernatants were collected and centrifuged for 10 min at 15,000 rpm. Two volumes of 0.5 N tri-n-octylamine in CH_2_Cl_2_ were added and again centrifuged for 3 min at 15,000 rpm; the supernatant was then analyzed using HPLC system with ODS reverse phase column (250×4.6 mm, 5 μm; Phenomenex), under the analytical method with eluent A (10 mM KH_2_PO_4_, 10 mM TBA-OH, 0.25% methanol: 50 mM KH_2_PO_4_, 10 mM TBA-OH, 30% methanol = 6:4, pH 6.7), and eluent B (50 mM KH_2_PO_4_, 10 mM TBA-OH, 30% methanol, pH 6.7) at a solvent flow rate 0.80 ml/min in a 175-min gradient elution as follows: B 1–23% from 0 to 20 min, B 23–45% from 20 to 130 min, B 45–99% from 130 to 150 min, B 99-99% from 150 to 160 min, B 99-1% from 160 to 160.1 min, and B 1-1% from 160.1 to 175 min.

### In vivo survival following introduction of xenografts

KM20C cell suspensions were prepared from *in vitro* cell culture and injected into the peritoneal cavity at a volume of 1×10^7^ cells on day 0. Drug treatment was started the next day (day 1). TAS-102 (150 mg/kg/day) was orally administered twice daily for 28 days, and S-1 (8.3 mg/kg/day) was also orally administered once daily for 28 days. The median increase of life span (ILS) was calculated as a survival index according to the following formula: ILS (%) = [(median survival time of treated group)/(median survival time of control group) - 1] × 100. The difference in the survival period distribution among groups was analyzed using the log-rank test.

### Ki-67 staining and morphological analysis

All reagents and antibodies were obtained from Dako, Tokyo, Japan. Heat-induced antigen retrieval was performed in Target Retrieval Solution with an autoclave at 120°C for 15 min. Endogenous peroxidase activity was inhibited with Peroxidase-Blocking Solution at room temperature (RT) for 5 min. The sections were then incubated at RT with the anti-Ki-67 mouse mono-clonal antibody (clone MIB-1; 1:150 dilution). The sections were washed in wash buffer and incubated with EnVision at RT for 30 min. The reaction products were visualized with a liquid diaminobenzidine substrate. The nuclei were lightly counterstained with hematoxylin.

### Statistical analysis

All statistical analyses were performed using SAS (SAS Institute, Cary, NC, USA). A P-value <0.05 was considered to indicate a statistically significant result.

## Results

### Comparison of FTD and FdUrd effects on cell growth and TS activity

We first compared the degree of growth inhibition and TS inhibition elicited by FTD and FdUrd following 24 and 72 h drug exposures. Table I shows the IC_50_ values derived from cell growth assays under these conditions. The cytotoxicity of FdUrd was 40.7-fold lower after the 24-h treatment when compared with 72-h treatment. Notably, the magnitude of this difference with respect to duration of drug exposure was much lower (2.7-fold) in the case of FTD treatment.

**Figure 1:**
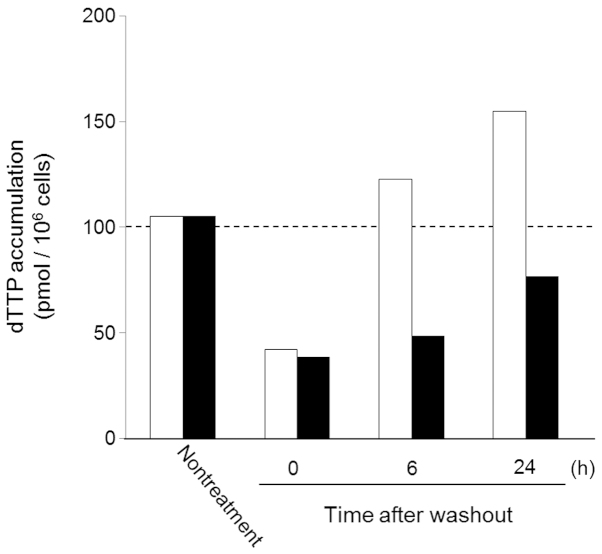
dTTP accumulation in HeLa cells after washout of FTD and FdUrd. Following 24-h FTD or FdUrd treatment, the drug was washed out and dTTP was measured by HPLC analysis as described in Materials and methods. Open and closed columns show dTTP accumulation by FTD and FdUrd treatment, respectively. The doses of FTD (2.5 μM) and FdUrd (0.01 μM) used were the IC_50_ values for the 72-h exposure. FTD, trifluridine; FdUrd, fluorodeoxyuridine.

Both FTD and FdUrd can inhibit TS (15). We therefore compared the amount of dTTP accumulation in HeLa cells after treatment with cytotoxic concentrations of FTD and FdUrd as an index of the TS inhibitory effect (16) (Fig. 1).

The dTTP levels in HeLa cells were markedly decreased after a 24-h treatment with either FTD or FdUrd. The dTTP pools did not reach pre-treatment levels following washout of FdUrd. In contrast, the dTTP pool in HeLa cells was restored to untreated levels between 6 and 24 h following washout of FTD. These results indicate that FTD-dependent inhibition of TS is reversible, and we therefore infer that inhibition of TS is not the major mechanism by which FTD exerts its cytotoxic effect.

### Comparison of incorporation of FTD and other nucleoside analogs into DNA

**Figure 2:**
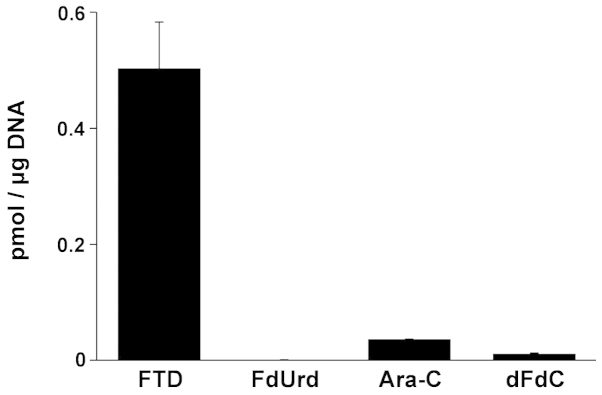
DNA accumulation of various antitumor nucleosides. NUGC-3 cells were treated for 4 h with each drug, and the amount that was incorporated into DNA was measured. The values are the means and the error bars are SD (n=3). The IC_50_ values of each drug after a 72-h treatment were chosen for this experiment (FTD 2.6 μM, FdUrd 0.10 μM, Ara-C 0.22 μM and dFdC 0.0045 μM; data not shown). FTD, trifluridine; FdUrd, fluorodeoxyuridine; Ara-C, cytarabine; dFdC, gemcitabine.

We next compared the DNA incorporation level of FTD to that of other nucleoside analogs, namely, FdUrd, Ara-C and dFdC, as they are known to be incorporated into DNA (17). Incorporation of tritium-labeled nucleosides at concentrations that induced the same level of cytotoxicity was allowed to proceed for 4 h (Fig. 2). The amount of FTD incorporation (0.50±0.05 pmol/μg DNA) was significantly higher than that of Ara-C 0.04±0.0001 pmol/μg DNA, dFdC 0.01±0.01 pmol/μg DNA and FdUrd 0.001±0.00004 pmol/μg DNA.

### Comparison of the in vivo antitumor activity, DNA incorporation level and dUMP accumulation following delivery of FTD via oral or continuous infusion routes

To translate our *in vitro* findings to a more relevant model, FTD was administered by oral and continuous infusion routes at either the maximal tolerated dose (MTD) or 2/3X MTD for 14 days of treatment.

**Figure 3:**
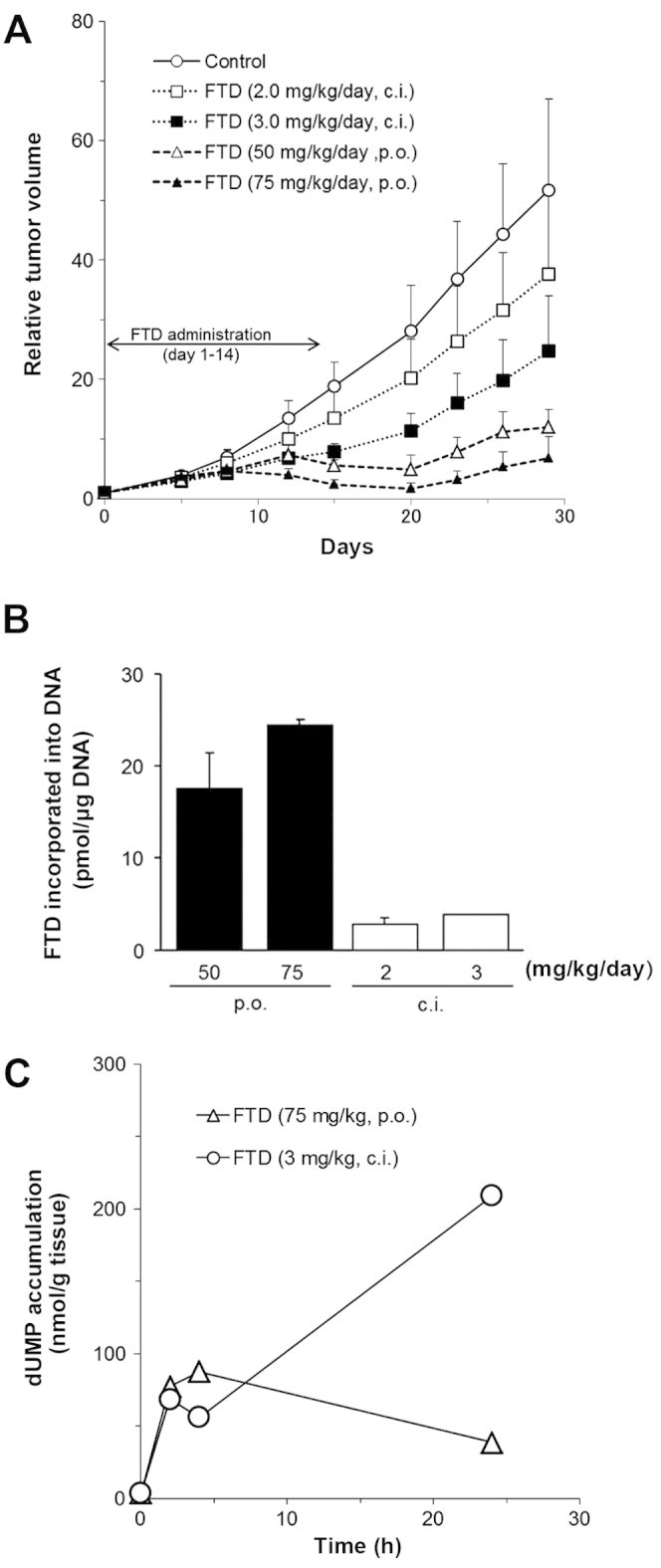
*In vivo* analysis of FTD accumulation and activity in xenograft models. FTD was administered by daily oral administration or continuous infusion for 14 day to mice subcutaneously implanted with human breast cancer MX-1. (A) Growth curve of xenografts. The tumor volume was measured twice a week and values indicate the means ± SD of the RTV (n=6–7). (B) The amount of FTD incorporated into DNA extracted from MX-1 was measured using HPLC analysis. Tumor for analysis of FTD accumulation was corrected at day 7. The values indicate the means ± SD (n=3). (C) The dUMP levels extracted from MX-1 were also measured using HPLC analysis. FTD was administered by oral administration at 0 h or continuous infusion from 0–24 h. FTD, trifluridine; dUMP, deoxyuridine monophosphate.

As shown in Table II, considerably higher doses of FTD were tolerated when the drug was administered orally rather than as a continuous infusion. For example, the change in body weight (−8.8±11.1%) with 50 mg/kg/day of oral FTD was similar to the change (7.9±12.0%) induced by 2 mg/kg/day of infusion FTD. Additionally, oral administration of FTD had a stronger antitumor effect than continuous infusion of FTD at the MTD (Table II). As an example, 50 mg/kg/day FTD oral administration gave an IR of 70.8% whereas 2 mg/kg/day FTD continuous infusion produced an IR of only 28.7%. Slight tumor shrinkage and tumor growth suppression was observed after FTD oral administration (Fig. 3A). Consistent with the above results, the amount of FTD incorporated into the DNA of xenograft tumor cells was higher after oral administration (p.o., 50 and 75 mg/kg/day FTD, 17.34±3.90 and 24.23±0.50 pmol/μg DNA respectively; c.i. 2.0 and 3.0 mg/kg/day FTD, 2.56±0.66 and 3.68±0.02 pmol/μg DNA, respectively). As an index of TS activity, we also evaluated dUMP accumulation in tumor tissues, as its levels increase when TS is inhibited (18). As shown in Fig. 3C, dUMP levels increased during continuous infusion treatment. The increase reached high level following continuous infusion treatment (208.9 nmol/g). On the other hand, the dUMP level after oral administration increased transiently, and reverted to almost basal level (39 nmol/g) at 24 h.

### Relationship between TAS-102 antitumor activity and the amount of FTD incorporation into tumor DNA

**Figure 4:**
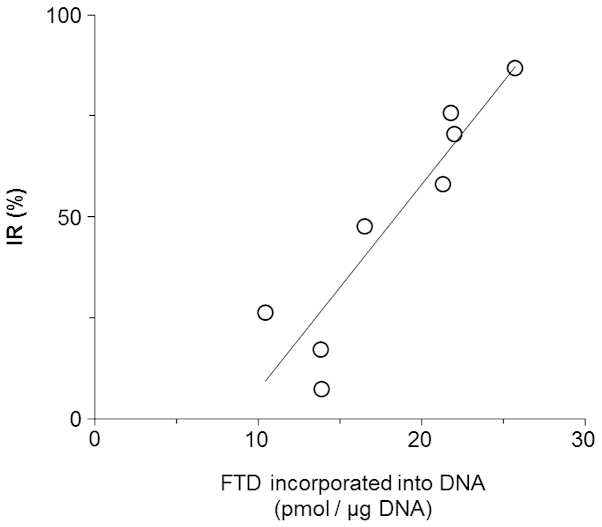
Relationship between the antitumor activity of TAS-102 and the amount of FTD incorporated into DNA. The amount of FTD incorporated into DNA in each tumor sample is plotted on the horizontal axis, and the IRs of each antitumor study is plotted on the vertical axis. All data are shown in Table III. A positive correlation was observed between the amount of FTD incorporated into the DNA of tumor cells and the antitumor effect of TAS-102 (Pearson correlation coefficient r=0.92, R^2^=0.84, P=0.0013). FTD, trifluridine; IRs, inhibition rates.

Since inhibition of TS did not appear to correlate with the antitumor activity of FTD, we hypothesized that direct interaction of the drug with DNA may contribute to its mechanism of action. TAS-102 (150 mg/kg/day) was orally administered to mice harboring xenografts in two daily doses for 14 days (Table III). This regimen was effective against xenografts derived from a broad range of tumor cell types. Moreover, inclusion of HPLC analysis at day 7 revealed a positive correlation between the antitumor activity of TAS-102 and the amount of FTD incorporated into DNA (Pearson correlation coefficient r=0.92, R^2^=0.84, P=0.0013 by Pearson correlation coefficient test, Fig. 4).

### FTD incorporation into DNA of a colon cancer-derived xenograft following TAS-102 treatment

**Figure 5:**
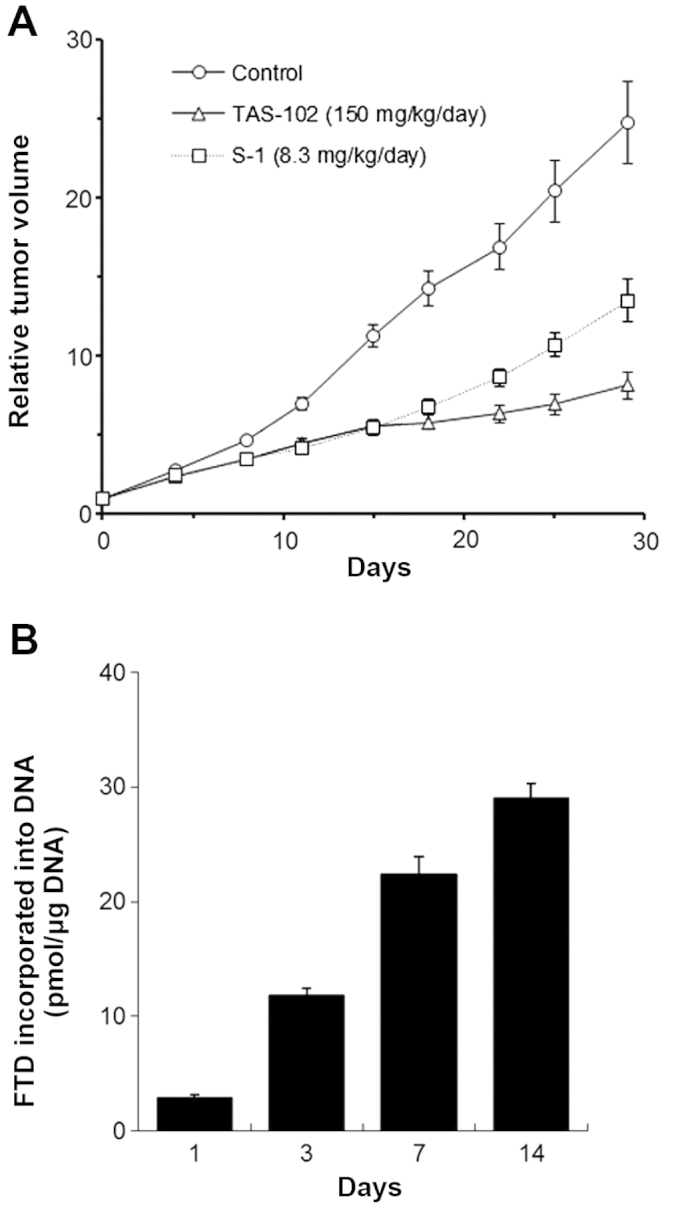
Antitumor activity of TAS-102 and S-1 and the amount of FTD incorporation into colon cancer xenograft DNA. TAS-102 (150 mg/kg/day) was administered twice daily for 14 days. S-1 (8.3 mg/kg/day) was administered once daily for 14 days. (A) Open circles, triangles and squares indicate control, TAS-102 and S-1 groups, respectively. (B) Amount of FTD incorporated into DNA following TAS-102 treatment. The values are the means ± SD (n=2 to 3). FTD, trifluridine.

To investigate the kinetics of FTD incorporation into DNA, we followed the process during TAS-102 treatment of mice harboring KM20C human colon cancer xenografts (Fig. 5A and B). TAS-102 treatment (150 mg/kg/day, twice daily for 14 days) was compared to oral administration of S-1 once daily for 14 days. TAS-102 and S-1 had similar antitumor effects (IR of 50.2 and 51.4%, respectively). FTD accumulated in the DNA of KM20C xenografts in a time-dependent manner throughout the administration of TAS-102. Compared to S-1 treatment, the growth suppressive effect of TAS-102 appeared more sustained (Fig. 5A).

### TAS-102 prolongs survival in mice bearing human colon cancer xenografts

**Figure 6:**
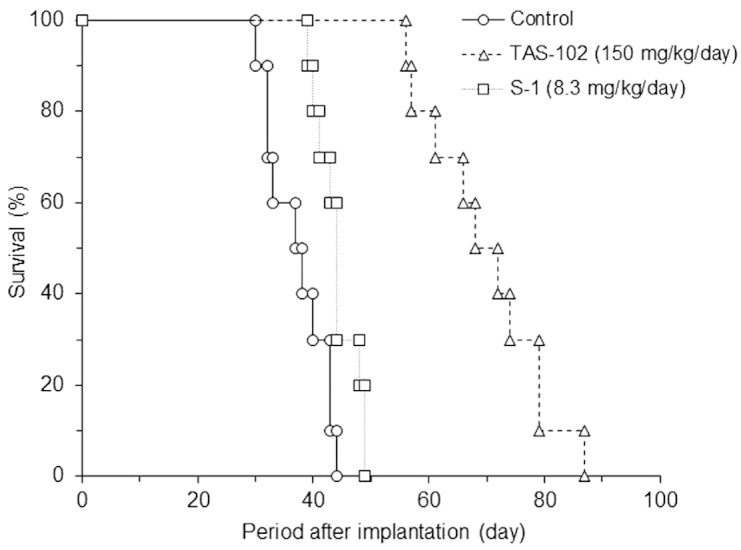
TAS-102 prolongs the survival of mice harboring colon cancer xenografts. KM20C colon cancer cells were implanted into the peritoneal cavity of nude mice at a volume of 1×10^7^ cells on day 0. All compounds were administered from day 1 to 28. TAS-102 (150 mg/kg/day) was administered twice daily. S-1 (8.3 mg/kg/day) was administered once daily. Open circles, triangles and squares indicate control, TAS-102 and S-1 groups, respectively.

To determine whether TAS-102 could prolong survival, we used mice implanted with KM20C xenografts (Fig. 6). The median survival time of control, TAS-102 and S-1 administered group was 38, 70 and 44 day respectively, and the ILS of TAS-102 and S-1 were 86.7 and 17.3%. TAS-102 significantly prolonged the survival of mice when compared to control (P<0.01), S-1 (P<0.01) as determined by the log-rank test.

### Ki-67 staining and morphological analysis

**Figure 7:**
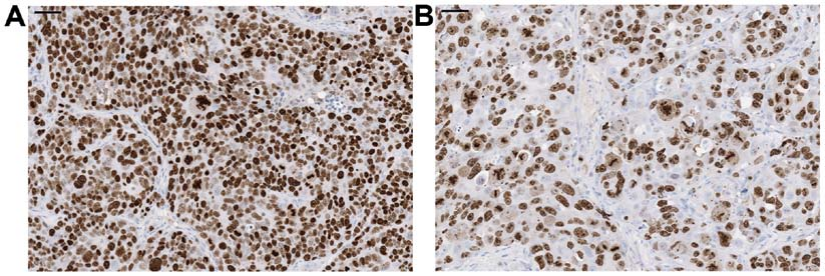
Ki-67 staining and morphological analysis for MC-2 treated for 14 days with TAS-102. TAS-102 (150 mg/kg/day) was administered to mice harboring MC-2 xenografts twice daily from day 1, and control (A) and TAS-102-treated samples (B) were collected on day 14. Ki-67 staining was performed as described in Materials and methods. Left upper bar indicates 50 μm.

To further characterize the growth suppressive activity of TAS-102 *in vivo*, we performed immunohistochemical staining for Ki-67 (a marker of proliferating cells), as well as morphological analysis (Fig. 7A and B). Tumor samples were collected from xenografts following a 14-day TAS-102 treatment. Ki-67 staining intensity was markedly decreased in most cells. Notably, we also observed a swelling of nuclei in the tumors treated with TAS-102.

## Discussion

FTD exhibits two major cellular activities, namely, inhibition of TS and incorporation into DNA. In the present study, we focused on determining which of these two activities plays the major role in the antitumor activity of TAS-102.

We first noted that both short- and long-term FTD treatments are cytotoxic; however, TS activity was only sustained by long-term treatment. In contrast, even short-term FdUrd treatment was sufficient to block TS activity, whereas the cytotoxicity of short-term FdUrd was diminished. This is likely since fluorodeoxyuridine monophosphate (FdUMP) inhibits TS by forming a covalent bond, whereas FTD monophosphate (F_3_dTMP) inhibits TS in a reversible manner (15), and short-term TS inhibition is not sufficient to exert cytotoxicity. We suggest, therefore, that the growth inhibition after FTD washout is likely due to DNA incorporation rather than TS inhibition. There are reports that FdUrd can be incorporated into DNA (19). However, fluorodeoxyuridine triphosphate (FdUTP) is efficiently degraded by deoxyuridine 5′-triphosphate diphosphohydrolase (dUTPase) (20). In the present study, we confirmed that the amount of FTD incorporation into DNA was much higher than FdUrd and other antitumor nucleosides. We thus infer that FTD and its cellular derivatives may be less prone to pathways that would otherwise reduce their ability to incorporate to DNA.

Secondly, both the amount of FTD incorporation into DNA and antitumor activity was higher, yet the TS inhibitory activity was lower, when the drug was orally delivered. We infer that persisting low concentrations of FTD following continuous infusion block DNA synthesis via inhibition of TS, and this leads to a small amount of FTD incorporation into DNA, and unwanted side-effects. Coincidentally, little amount of FTD incorporates into DNA, since DNA synthesis is inhibited by TS persisting inhibition. By contrast, oral administration does not appear to inhibit TS chronically, and, therefore, DNA synthesis is less affected and FTD incorporates into DNA during DNA synthesis. It is reported that intracellular F_3_TTP concentrations reach a plateau after 8 h, and decline after washout (21). From these considerations, we suggest that daily oral administration of FTD could be a suitable regimen for antitumor treatments.

A significant caveat is that FTD is degraded when administered orally. Indeed, the activity of TP in the liver of monkeys and humans is high, which can negatively affect systemic drug concentrations. In the present study, a positive correlation was observed between the level of FTD incorporated into tumor cell DNA and the antitumor activity of TAS-102 toward various xenografts. This relationship suggests that FTD incorporation into DNA is the major mechanism underlying the antitumor activity of TAS-102. Notably, the antitumor effect of TAS-102 is sustained after administration (Fig. 5A), and a similar result was observed following FTD oral administration (Fig. 3A). This persistent antitumor activity likely accounts for the ability of TAS-102 to prolong the survival of xenograft-bearing mice. FTD is incorporated in place of thymidine bases in DNA (19), and this only slightly destabilizes the DNA duplex (22); it may alter DNA-protein interactions and cause nuclei swelling due to nucleosomal abnormality. Thus, the ability of FTD to accumulate in the DNA of tumor cells without causing immediate catastrophic consequences may allow it to affect tumor cells in a chronic and persistent manner. Bijnsdorp *et al* (23) stated that mechanisms by which FTD induces cell death are distinct from those elicited by 5-FU. It was also reported that TAS-102 monotherapy significantly improved overall survival for patients with mCRC and who were insensitive to fluoropyrimidine-based standard therapies (10). Further analyses are needed to elucidate the mechanisms responsible for the cytotoxicity of FTD following its incorporation into DNA.

In conclusion, we suggest that, due to its unique mechanism of action, which is distinct from that of existing 5-FU derivatives, TAS-102 has potential as a potent therapeutic option for malignancies that are insensitive to fluoropyrimidine-based therapy.

## Figures and Tables

**Table I:** Growth inhibitory effect of the FTD and FdUrd on HeLa cells following continuous or intermittent treatment.

Compounds	Treatment duration (h)	IC_50_^a^ (μM)	Ratio^b^
FTD	24	13.4	2.7
	72	4.9	
FdUrd	24	37.1	41.2
	72	0.9	

In the 72-h treatment experiment, HeLa cells were treated with FTD or FdUrd continuously for 72 h. In the 24-h treatment experiment, the drug was washed out at 24 h and replaced with drug-free medium for a further 48 h.

a Concentrations that gave 50% growth inhibition;

b the IC_50_ ratio of 24- and 72-h treatment.

FTD, trifluridine; FdUrd, fluorodeoxyuridine.

**Table II:** Antitumor efficacy of FTD in human breast cancer xenografts following oral administration or continuous infusion.

Treatment	Administration route	RTV^a^ (mean ± SD)	IR^b^ (%)	BWC^c^ (%, mean ± SD)
Control	-	18.9±3.9	-	17.6±8.1
50 mg/kg/day FTD	p.o. (b.i.d.)	5.5±1.0^e^	70.8	−8.8±11.1
75 mg/kg/day FTD	p.o. (b.i.d.)	2.4±0.8^e^	87.5	−23.9±4.4
2.0 mg/kg/day FTD	c.i.	13.5±4.7^d^	28.7	7.9±12.0
3.0 mg/kg/day FTD	c.i.	7.9±1.3^e^	58.2	−13.6±9.1

FTD was administered for 14 days via each administration route.

a Relative tumor volume (RTV) on day 15;

b tumor growth inhibition ratio (IR) on day 15;

c relative body weight change (BWC) between day 0 and 15;

d P<0.05,

e P<0.01 according to Dunnett’s test;

p.o., per os; b.i.d., bis in die; c.i., continuous infusion. FTD, trifluridine.

**Table III:** Antitumor efficacy of TAS-102 and DNA incorporation of FTD in xenografts derived from various cell types.

Tumor	Origin	IR^a^ (%)	FTD incorporated into DNA
DLD-1	CRC	7.3	13.9
HT-29	CRC	17.2	13.8
SW480	CRC	26.4	10.4
HCT116	CRC	47.6	16.5
KM20C	CRC	70.5	22.0
Lu-134	SCLC	75.8	21.8
MC-2	BC	58.0	21.3
MX-1	BC	86.9	25.7

TAS-102 was orally administered twice daily for 14 days at 150 mg/kg/day. Tumor for analysis of FTD accumulation was collected at day 7.

a Tumor growth inhibition ratio on day 15.

CRC, colorectal cancer; SCLC, small cell lung cancer; BC, breast cancer; FTD, trifluridine.
